# The Use of BEREP4 Immunohistochemistry Staining for Detection of Basal Cell Carcinoma

**DOI:** 10.1155/2017/2692604

**Published:** 2017-12-31

**Authors:** Anthony Paulo Sunjaya, Angela Felicia Sunjaya, Sukmawati Tansil Tan

**Affiliations:** Faculty of Medicine, Tarumanagara University, Jl. Letjen S. Parman No. 1, Jakarta 11440, Indonesia

## Abstract

Basal Cell Carcinoma (BCC) is the most common type of malignant cancer found in the world today with a 3–10% increase in incidence each year. The American Cancer Society reported that 8 out of 10 patients with skin cancer are suffering from BCC with over 2 million new cases each year. BCC needs to be detected at the early stages to prevent local destruction causing disabilities to patients and increasing treatment costs. Furthermore, BCC patients who have undergone surgery are still at risk for recurrence, especially when the surgery performed fails to remove all the BCC cells, even when conventional histopathological testing after surgery has reported a surgically free margin. This review aims to evaluate studies on the use of BerEP4 immunohistochemistry staining on pathological sections of various types of BCC as well as its shortfalls. BerEP4 is a monoclonal antibody which detects specific epithelial-glycoprotein-adhesion-molecules (EpCAM) found on BCC cells. Various studies have shown that BerEP4 has a high sensitivity and specificity in detecting only BCC cells. The use of BerEP4 immunohistochemistry testing for the routine examination of cases of BCC is expected to be able to increase and improve early diagnosis as well as prevent recurrence after surgery.

## 1. Introduction

First discovered by Jacob et al. in 1827 and named* rodents ulcer*, basal cell carcinoma (BCC) is a type of nonmelanocytic malignant skin cancer. BCC arises from the basal cells of the epidermis and hair follicles. It is currently the most commonly found skin cancer in humans [1, 2].

The prevalence of BCC increases at a rate of 3–10% each year. The American Cancer Society (2012) reported that 8 out of 10 patients with skin cancer are suffering from BCC with more than 2 million new cases each year. BCC is also one of the main types of skin cancer found in Indonesia, constituting 36.67% of skin cancer patients, and more prevalent than any other types of skin cancers such as squamous cell carcinoma (11.4%) and melanoma (0.59%) [3–6].

The prevalence of BCC is also found to be twice more in men than in women, with increasing prevalence with older age. A higher incidence (more than 100-fold) of BCC was found in patients aged 55–70 years compared to those below 20 years old. BCC is rarely found in people under the age of 40 years, although, currently, the incidence in youth continues to rise due to increasing advances in the early diagnosis of BCC [3, 7].

BCCs are caused by frequent exposure to ultraviolet radiation, most commonly ultraviolet spectrum B (UV-B) with a wavelength of 290–320 nm, inducing mutations in tumor suppressor genes. Moreover, UV-B radiation damages the DNA and affects the immune system. In the long run, these genetic changes can cause neoplasms. Mutations in the p53 tumor suppressor gene have been found in approximately 50% of BCC cases. As a result, BCCs are mostly found in body parts frequently exposed to sunlight such as the face, scalp, and neck [1, 3].

## 2. Types of BCC

BCCs can be classified into several different types based on their morphologies as follows: nodular BCC, cystic BCC, infundibulocystic BCC, morpheaform/cicatricial BCC, infiltrative BCC, micronodular BCC, superficial BCC, pigmented BCC, rodent's ulcer, fibroepithelioma of Pinkus, polypoid BCC, pore-like BCC, aberrant BCC, and solitary BCC. These various types of BCC exhibit different biologic behaviours with varying clinical, pathological profiles and prognoses [8].

The most aggressive BCCs are of the infiltrative and morpheaform type. Aberrant BCCs refer to those which are found in odd sites such as the scrotum, perineum, and axilla without direct or apparent exposure to carcinogenic factors such as radiation, arsenic, and chronic ulceration, whereas superficial BCCs are commonly found in immunocompromised hosts such as those with an underlying Human Immunodeficiency Virus (HIV) infection and patients with transplants [8].

## 3. Diagnosis

At present, histopathological examination using Hematoxylin and Eosin (H&E) is the gold standard to confirm the clinical and dermoscopic diagnosis of BCC. However, histopathological examination is not always able to accurately diagnose and distinguish some types of BCC morphologically similar to other types of carcinomas such as peripheral ameloblastoma or distinguish BCC with basosquamous carcinoma whose treatment should differ medically from BCC due to its higher metastasis capabilities [13–16].

## 4. Therapy and Prognosis

BCC has locally invasive properties as well as low metastatic ability and can easily be treated by surgical excision, provided it is diagnosed at an early stage. However, as they are often asymptomatic, patients often seek treatment in the later stages wherein the BCC has infiltrated the surrounding tissues. It has been found that the average duration from onset to diagnosis of BCC is 37.1 months. By this time the BCC would have been greater than 2 cm, increasing the risk of recurrence after therapy [2, 17].

Late diagnosis and management of BCCs can cause not only disability (scarring the appearance) but also functional impairments to vision, hearing, taste, and smell depending on the site of occurrence. Furthermore, later forms of BCCs pose greater difficulty in treatment. After therapy, these patients have a higher risk for recurrence especially when the results of histopathological examination on incision edges are positive for BCC cells. Even in those where histopathological examination of the edge of the incision has been declared free of BCC cells after surgery, the potential for recurrence remains. Recurrent BCCs area is associated with worse prognosis and a greater risk of metastasis [2].

Although improvements have been made in the prognoses of patients with BCC, recurrence still occurs due to the remnants of BCC cells that are not removed or detected by histopathological examination with H&E, the current gold standard. Research by Mosterd et al. (2011) showed that identification of primary BCC subtypes using conventional histopathological examination (H&E) has a diagnostic accuracy of 80.7%. Moreover, diagnosing recurrent BCC is even much more difficult due to its disjointed growth as a result of the formation of scar tissue [2, 18].

Therefore, as an adjunct to standard diagnostic techniques, an additional diagnostic test is needed that can detect BCC at its early stages as well as detect BCC cells that are not removed after surgical excision therapy. This test would need to be able to distinguish BCC from other carcinoma and skin disorders like squamous cell carcinoma, ameloblastoma, actinic keratosis, and so on. With increasing prevalence of BCC as a result of increased radiation due to global warming and an aging world, the issue of accurately diagnosing BCC is becoming more important than ever. This review aims to evaluate the potential of BerEP4 immunohistochemistry staining in detecting basal cell carcinoma at an early stage and preventing the recurrence of basal cell carcinoma after therapy.

## 5. Materials and Methods

The databases searched to obtain the articles included MEDLINE Full Text, Pubmed, Science Direct, Pro Quest, SAGE, Taylor and Francis Online, Google Scholar, High Wire, and Elsevier Clinical Key. The search strategies used included availability of full text written in English from 1 January 2000 till 31 December 2016.

Keywords used were “basal cell carcinoma” AND “BerEP4” AND “immunohistochemistry or synonyms.” When multiple articles for a single study were found, the most recent publication was used. Relevance of studies was assessed by using an approach based on title, abstract, and full text. Studies were included if they were original studies of any design.

## 6. BerEP4 Immunohistochemistry

Immunohistochemistry is a technique that can be used to locate cell antigens, tissue, amino acids, proteins, and infectious agents found on specific cells. Presently, immunohistochemistry has become an important tool in medical research and serves as a companion in verifying differential diagnosis which cannot be determined by conventional analysis using H&E staining [19].

Morphologically BCC includes a group of intradermal tumors with cellular characteristics that resemble components of undifferentiated epidermal basal cells. BCCs are derived from stem cells or progenitor cells in the basal membrane enabling BCC to come in various forms and appearances. Some studies have shown that the majority of BCCs are monoclonal tumors and BCCs which differ anatomically may sometimes derive from the same cell origin [5].

All epithelial cells expressed cell adhesion molecules (CAMs) which can be subdivided into 4 families: cadherins, selectins, integrins, and immunoglobulins (Ig)-like-CAMs. Additionally, several CAMs can also be found that do not belong to these “classical” families of adhesion molecules, one of them known as EpCAM [20–22].

The study by Maetzel et al. (2009) showed that EpCAM has a role in cell signaling, proliferation, adhesion, migration, and differentiation. EpCAM was overexpressed in epithelial progenitor cells, carcinoma cells, and cancer triggering cells. Studies have found that EpCAM expression is high in proliferating cells and low in differentiated cells. It was also found to work as an oncogenic signaling molecule through the Wnt signaling pathway. The mechanism of EpCAM induced proliferation in cancer cells has been shown to involve regulated intracellular membrane proteolysis (RIP). EpCAM was found to be a cell surface glycoprotein highly expressed in nonsquamous epithelial cancers and at lower levels in normal simple epithelia [23, 24].

The above epithelial-specific antigen is also found in carcinomas of other organs such as ovarium, colon, prostate, stomach, and lung carcinoma. Currently, anti-EpCAM antibody has been available for use for histopathologic diagnosis and therapy in humans. BerEP4 is an anti-EpCAM antibody and has proven to be a sensitive marker towards BCC [25–27].

BerEP4 is a monoclonal antibody that can be used as a marker of BCC. It works by detecting the EpCAM antigen, a transmembrane epithelial glycoprotein cell adhesion molecule found in humans. BerEP4 is made from human mammary as well as mice carcinoma cell lining (MCF-7). This marker, which is a mouse IgG antibody, can detect glycopeptides with masses of 34 kD, 39 kD, and 40 kD [16, 24].

## 7. Study Results on BerEP4 Use in Detecting BCC

One of the first studies to evaluate the use of BerEP4 in detecting BCC cases was performed by Beer et al. (2000). It was reported that BerEP4 was able to detect all 39 BCC samples used in this study. The same result was obtained by Ishida et al. (2008) with studies being performed on 20 BCC samples. Krahl & Sellheyer (2007) also conducted tests with BerEP4 on 28 sclerosing and infiltrative BCCs. All 28 BCCs came with varying degrees of positive results, in which 27 have a degree of moderate to strong positives and one weak positive [29, 30, 37].

Another study conducted by Karahan et al. (2006) on 20 specimens of BCC also reported that BerEP4 was able to detect all the BCC specimens. Further research using BerEP4 immunohistochemistry consistently gave similar results; the study by Dasgeb et al. (2013) using BerEP4 to distinguish 24 BCC specimens with 88 common skin neoplasms, namely, trichoepithelioma, actinic keratosis, squamous cell carcinoma in situ, squamous cell carcinoma, seborrheic keratosis, lichen planus like keratosis, nevi, hemangioma, inverted follicular keratosis, sebaceous adenoma, and Merkel cell carcinoma (MCC) from dermatopathology files, also showed that it was able to diffusely stain all BCC sections. However, all trichoepitheliomas and MCCs were also diffusely stained while 1 squamous cell carcinoma and 1 sebaceous adenoma showed weak staining. The false positive rate was at 15% [16, 24].

Further immunohistochemistry tests were also conducted by Sellheyer, et al. (2013) to test the usefulness of BerEP4 in diagnosing morpheaform BCCs. The results showed that, among 17 specimens, 16 showed an immunological reaction of more than 75% while the other one showed an immunological reaction at over 25%. Ansai et al. (2012) in their study of 31 BCC cases, 18 cases of nodular type, 9 cases of superficial type, and 4 cases of morpheaform type, also reported a 97% sensitivity of BerEP4 in detecting BCC cells [34, 35].

**Figure 1:**
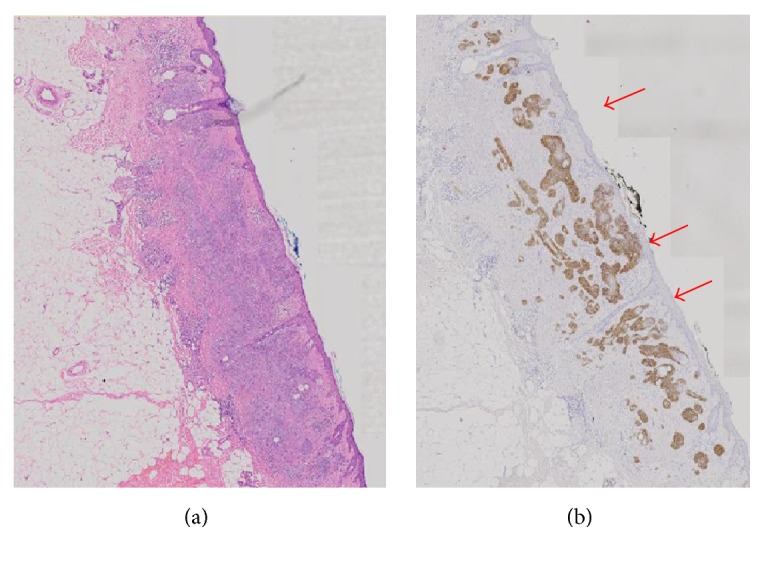
Comparison H&E stain (a) with BerEP4 immunohistochemistry staining (b) on BCC pathological sections (10x magnification) [9].

Our previous study on BerEP4 use conducted by Tan & Sunjaya (2016) on 23 specimens with 394 microlesions also showed 100% positive results with 100% sensitivity and specificity. In the study, BerEP4 has been proven to detect and provide positive results in the early stages of BCC consisting of only several cells [9] (Figure 1).

### 7.1. Differentiating BCC from Squamous Cell Carcinoma (SCC)

One of the main advantages of BerEP4 is the ability to distinguish BCC from squamous cell carcinoma (SCC) with high sensitivity and specificity. Both BCC and SCC arise from the epidermal layer with similar predilection on areas of the skin exposed to the sun; hence they both often show a similar clinical profile. Although SCCs which are similar in profile with BCCs rarely spread, they still do so more often than BCCs. As a result, these 2 disorders must be accurately differentiated to prevent misdiagnosis and incorrect treatment. Data from various studies compiled by Sellheyer et al. (2013) showed that all 75 cases of SCC stained with BerEP4 gave negative results. The same result was reported by Karahan et al. (2006) and Dasgeb et al. (2013). The above results are also supported by research performed by Ozawa et al. (2004) which showed that BerEP4 antigens are not found in normal keratinocytes and squamous cell carcinoma of the skin [16, 24, 34, 38].

Research by Mashhood et al. (2011) to find the sensitivity and specificity of BerEP4 in patients of Asian origin with SCC as control also reported that BerEP4 has a sensitivity and specificity of 100% [32]. These results do not differ much with the sensitivity and specificity calculated based on the summary of the studies on the usage of BerEP4 with SCC as control, showing the sensitivity of BerEP4 as 99.6% and specificity of BerEP4 as 99.2% with a positive predictive value of 99.6% and negative predictive value of 99.1% (Table 1).

### 7.2. Differentiating BCC from Basosquamous Carcinomas (BSC)

In addition, BerEP4 also have important uses in diagnosing basosquamous carcinomas (BSC), which are currently difficult to identify and diagnose definitively. Definitive diagnosis of BCC and BSC is required and of great importance due to their differences in prognosis and treatment. A study by Karahan et al. (2006) showed that the identification of BSC can be facilitated by BerEP4 staining. This can be due to the fact that BSC is a tumor similar to BCC with squamous differentiation and therefore has an immunohistochemical profile that is similar to BCC. The difference between the results of staining BSC and BCC lies in the extent of staining of the specimen, in BSC, patchy stains were found which only cover part of the tumor studied [16].

### 7.3. Differentiating BCC from Collision Tumors

**Figure 2:**
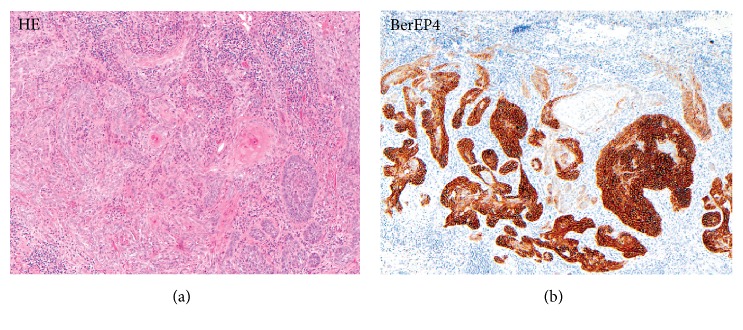
Comparison H&E stain with BerEP4 immunohistochemistry staining on a pathological section having BCC with squamous cell metaplasia. Only BCC cells are stained with BerEP4. (100x magnification) [10].

BerEP4 can also be used to distinguish collision tumors, a type of tumor that is a mixture of BCC and SCC. Areas of BCC will be strongly stained whereas areas of SCC will remain untainted by BerEP4 [16] (Figure 2).

### 7.4. Differentiating BCC from Basaloid Squamous Cell Carcinoma (bSCC)

While previous studies have reported that BerEP4 can differentiate BCC from BSCs and collision tumors, Linskey et al. (2013) reported that BerEP4 alone was unable to differentiate bSCC and BSC as both were stained by BerEP4, although the mean percentage of cells stained was significantly higher in BSC group compared to bSCC. In detecting bSCC, BerEP4 was reported to have a sensitivity of 60%, specificity of 44%, positive predictive value (PPV) of 65%, negative predictive value (NPV) of 39%, and false positive rate of 25% [10].

### 7.5. Differentiating BCC from Sebaceoma

Research conducted by Fan et al. (2007) to compare the reaction of BerEP4 in BCC and sebaceoma showed that out of the 25 sebaceoma specimens tested, 24 were negative while one showed a weak positive result (<10% tumor cells stained) with a false positive rate of 4%. Meanwhile, all tested nodular BCC specimens (51 specimens) were moderately or strongly stained, with never less than 20% of the tumor stained by BerEP4 [28].

### 7.6. Differentiating BCC from Microcystic Adnexal Carcinoma (MAC)

Other studies also showed that BerEP4 was able to distinguish BCC from microcystic adnexal carcinoma (MAC). Sellheyer et al. (2013) and Krahl et al. (2007) reported that BerEP4 was able to reliably differentiate MAC from BCC to the same extent as it distinguishes BCC from SCC. None of the MACs which were taken as control were stained in the study [29, 34].

### 7.7. Differentiating BCC from Bowen's Disease

**Figure 3:**
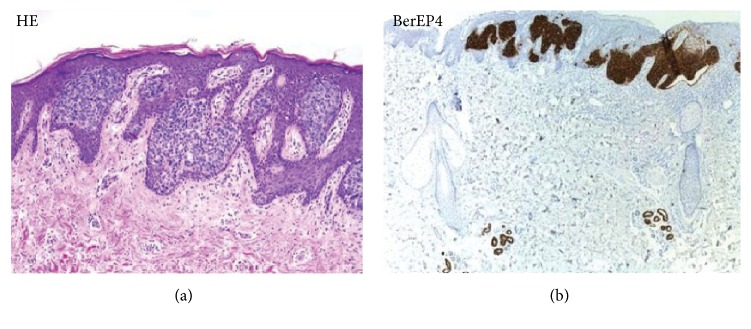
Comparison H&E stain (200x magnification) with BerEP4 immunohistochemistry staining (100x magnification) on superficial BCC pathological sections mimicking Bowen's Disease [11].

Conflicting results have been reported with regard to BerEP4 use in distinguishing BCC from Bowen's Disease (BD). BerEP4 was found to be able to differentiate BCC from BD with a specificity and sensitivity of 100% in a study by Ansai et al. (2012), although a recent study by Kogut et al. (2016) reported that BerEP4 expression is not always helpful in distinguishing BD from BCC with around 26% of BD's reacting positively with BerEP4 (at least 5% staining) [39] (Figure 3).

### 7.8. Differentiating BCC from Other Epithelial Skin Disorders

Actinic keratosis [36], seborrheic keratosis [35], poroma [35], lichen planus like keratosis [24], nevi [24], hemangioma [24], inverted follicular keratosis [24], squamous intraepithelial neoplasia (SIN) [24], and sebaceous adenoma/hyperplasia [24] are a wide variety of epithelial skin disorders that have been proven to give negative results on BerEP4 testing (Table 2).

In addition to the studies above, various other studies and case reports also showed that BerEP4 can detect even rare forms of BCC such as perianal BCC, intraoral BCC, and axillary BCC with high sensitivity and specificity. In many cases, BerEP4 use has been of great help in preventing misdiagnosis of the skin disorders. The results of these studies are summarised below.

### 7.9. Perianal BCC

**Figure 4:**
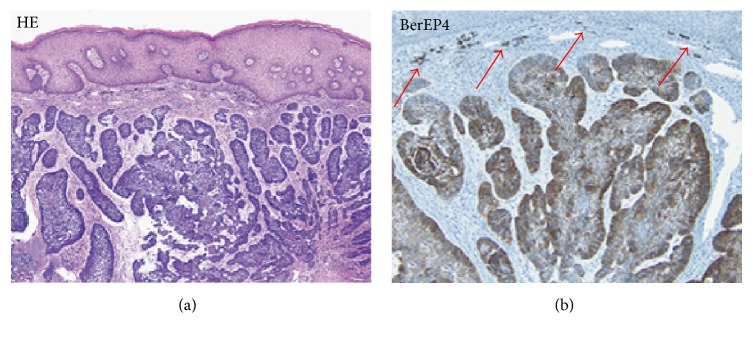
Comparison H&E stain with BerEP4 immunohistochemistry staining on perianal BCC pathological sections (20x magnification). Arrows show small groups of BCC cells difficult to differentiate with the H&E Stain [12].

A study by Patil et al. (2013) used BerEP4 markers (Dako, Carpinteria, CA, USA) to identify BCC located in the anal region. These carcinomas are rarely found and hard to distinguish from basaloid squamous carcinoma without the use of biopsy techniques. In this study, specimens consisted of 9 BCC and 15 basaloid squamous carcinomas. The results show that BerEP4 staining was able to diffusely stain all BCCs. Interestingly, 40% of the basaloid specimens were also stained by BerEP4 in this study. A case report by Kreuter et al. (2012) also reported that immunohistochemical staining with BerEP4 on perianal tumors specimens showed strong positive results [12, 40] (Figure 4).

### 7.10. Intraoral BCC

Intraoral BCC case reports obtained also show that the use of BerEP4 can help distinguish intraoral BCC from peripheral ameloblastomas, both of which possess similar histological features. Of the four reported cases of intraoral BCC, all were initially misdiagnosed as peripheral ameloblastoma due to its similarity. The tumor can only be confirmed as intraoral BCC after immunohistochemical examination with BerEP4 marker yielded a positive result [13, 15].

**Figure 5:**
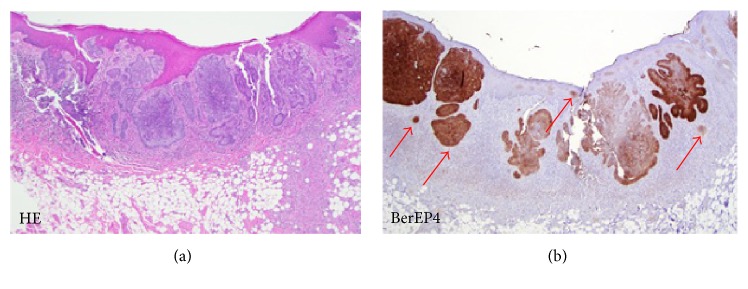
Comparison H&E stain with BerEP4 immunohistochemistry staining on intraoral BCC pathological sections (4x magnification). Arrows show small groups of basaloid cells difficult to differentiate with the H&E Stain [13].

Recent research conducted by Sook & Keun (2014) to compare protein expression between intraoral BCC and peripheral ameloblastoma detected with 50 different types of antisera also showed that intraoral BCC showed strong positive results against EpCAM [41] (Figure 5).

### 7.11. Axillary BCC

Due to the specificity and sensitivity of BerEP4 in detecting BCCs, some rare BCC cases can be ascertained and diagnosed as BCC only after BerEP4 immunohistochemical test results gave strong positive results. Axillary BCCs are rare as this region of the body is rarely exposed to the sun. Four cases of rare axillary, as well as one case of BCC who experienced postoperative recurrence and metastasis, have been diagnosed with the help of BerEP4 immunohistochemistry test [42, 43].

### 7.12. Metatypical and Granular BCC

Two metatypical BCC cases with a high rate of postoperative recurrence and metastasis risk [44, 45] as well as two cases of granular BCC found by Claassen et al. (2014) and Jedrych & Busam (2014) were also successfully diagnosed with the help of BerEP4 immunohistochemistry test [46, 47].

### 7.13. Eyelid BCC and in Epidermoid Cysts

Jakobiec et al. (2010) reported a case of BCC found in epidermoid cysts on the eyelids which was confirmed through BerEP4 immunohistochemistry test. Two years later, Kirzhner and Jakobiec (2012) retrospectively analysed 13 specimens of pigmented BCC specimens found in the eyelid. They reported that BerEP4 was found positive in all the lesions. BerEP4 was positive within the basaloid tumor cells but negative in metatypical and intratumoral melanocytes [33, 48].

## 8. BerEP4 Use after Surgery for Prevention of Recurrence

In accordance with the guidelines for handling BCC made by the British Association of Dermatologists, incision of small lesions (<20 mm) with clear boundaries with peripheral surgical margin of 3 mm will eliminate all BCC cells in 85% of cases, while a margin of 4-5 mm would eliminate all BCC cells in 95% of cases [49].

**Figure 6:**
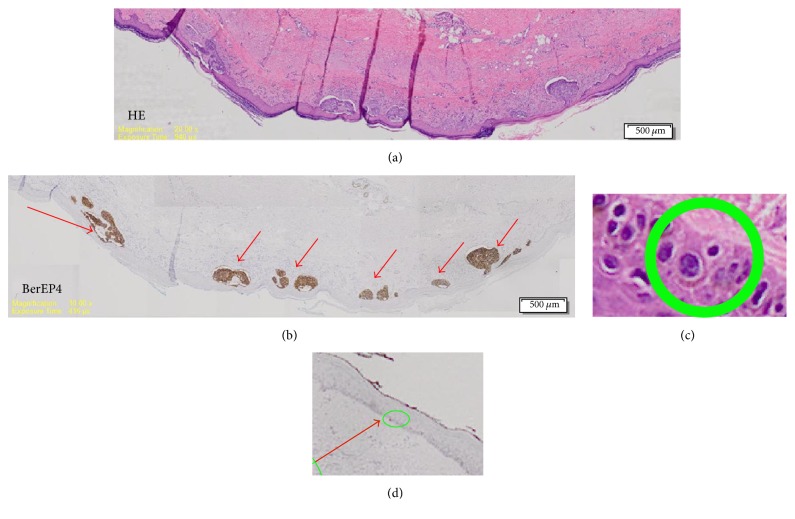
Comparison H&E stain with BerEP4 immunohistochemistry staining on BCC pathological sections with Multiple microlesions [9]. ((a) 20x magnification, (b) 10x magnification, (c) 100x magnification, (d) 10x magnification).

BCC patients are at risk for recurrence even after the incision edge has been declared free of BCC cells by H&E staining as several types of BCCs often have multiple microlesions which are spread out over the skin surface. These microlesions are difficult to detect and identify with normal staining as they are at times still a small group of cancer cells (Figure 6).

In a study by Kist et al. (1997), 27 BCC specimens (15 nodular BCC, 11 Morpheaform BCC, and 1 Adenoid BCC) were examined for remaining BCC cells after Mohs Micrographic Surgery. Specimens were stained with BerEP4 and the results showed BCC cells remaining at 2 specimens that had previously been checked by H&E staining and provided negative results. In addition, BerEP4 also provides enhanced visualization of the 13 specimens previously reported positive with H&E staining, especially on the BCC specimens of the morpheaform type [50].

Filho et al. (2008) conducted a study using BerEP4 testing on 20 BCC specimens to ensure the edge of the incision has been free of BCC cells after curettage treatment and two cycles of electrofulguration. The test resulted in 5 BCC specimens (25%) being positive for remaining BCC cells [31].

Various studies have consistently reported that BerEP4 has a sensitivity of 100% and gives positive results in all BCC specimens examined. Besides being able to deliver positive results in frequent BCC locations such as the neck and face, BerEP4 also provide positive results in frequently found subtypes such as morpheaform BCC and nodular BCC, as well as positive results in rarely found BCC such as the axilla, intraoral, perianal, and granular BCC.

## 9. BerEP4 Limitations

However, BerEP4 was found to be unable to distinguish BCC from trichoepithelioma, trichoblastoma, Merkel cell carcinoma (MCC), and basaloid squamous cell carcinoma (bSCC). One of the reasons for this is that trichoepithelioma, trichoblastoma, and BCC are all epithelial neoplasms of follicular germinative cell differentiation. To distinguish trichoepithelioma and trichoblastoma with BCC requires the use of other immunohistochemistry test using pleckstrin homology-like domain (PHLDA1), whereas a panel of BerEP4, cytokeratin (CK) 14, and CK17 is needed to reliably differentiate BCC and BSCs from bSCCs [10, 24, 34, 35].

MCC is a malignant cutaneous neoplasm with epithelial and neuroendocrine differentiation. Therefore MCCs are positive for epithelial and neuroendocrine markers but are negative for lymphoid and melanoma markers. So far no single immunohistochemical examination method has been found which can accurately detect MCCs; the diagnosis can only be confirmed through the use of several immunohistochemical markers to exclude microscopically similar tumors such as melanoma, small cell metastatic lung carcinoma (SCLC), and BCCs. A combination of CK20 which is highly sensitive for MCCs, together with thyroid transcription factor-1 (TTF-1) and CK7 which are expressed in SCLCs but consistently absent in MCCs as well as HBM45, NKI/C3, and S-100 markers, which are positive in melanoma but negative in MCCs, can be used to identify MCCs [24, 51–53].

## 10. Practical Implications

From the various studies using BerEP4 immunohistochemistry explained above, differences remain to be found with regard to the sensitivity and specificity of results which can occur due to the use of different brands of BerEP4, differences in the original staining of the specimens, old specimens being analysed (especially when the specimens derive from dermatopathology archives), differences in staining techniques, the tools used, and the stage of the lesion being diagnosed.

The results of the studies found, however, suggest that immunohistochemistry testing using BerEP4 has a high sensitivity and specificity. BerEP4 use is therefore recommended especially in patients suspected of having BCC with multiple microlesions, infiltrative BCC, BCCs with mixed pathologies, and BCCs located in rare sites like the axilla, perianal region, and so on. In addition, BerEP4 can also be used to identify tumor excision margins especially in patients with recurring BCC lesions.

## 11. Conclusions

Currently, immunohistochemistry testing has become an important adjunct and tool for differential diagnosis especially for studying parameters that cannot be viewed, defined, or analysed with conventional staining. BerEP4 is a monoclonal antibody which can detect the presence of EpCAM antigen found on BCC cells. It has been proven to be able to reliably detect BCCs of all types and even those in rare locations including intraoral, axillary, metatypical, and granular BCC.

BerEP4 has been found to be able to differentiate BCC from other cutaneous pathologies such as squamous cell carcinoma, basosquamous cell carcinoma, collision tumors, sebaceoma, microcystic adnexal carcinoma, ameloblastoma, epidermoid cysts, actinic keratosis, seborrheic keratosis, poroma, lichen planus like keratosis, nevi, hemangioma, inverted follicular keratosis, squamous intraepithelial neoplasia, and sebaceous adenoma/hyperplasia. However, BerEP4 was found to be unable to distinguish BCC from trichoepithelioma, trichoblastoma, Merkel cell carcinoma, and basaloid carcinoma. Conflicting results have also been reported regarding BerEP4 use in differentiating BCC from Bowen's Disease. Despite its limitations, studies have shown that BerEP4 has the advantage of being able to detect small amounts of BCC cells making it suitable for early detection of BCC as well as in detecting BCC mixed with other cutaneous pathology. Moreover, it was able to accurately identify the presence of BCC cells after therapy and therefore is expected to reduce the incidence of recurrence after therapy.

## Figures and Tables

**Table 1:** Sensitivity, specificity, positive predictive value, and negative predictive value of BerEP4 in detecting BCC with SCC as control.

Studies	BCC +	SCC + (control)
Karahan, et al. [16]	20/20	0/20
Fan, et al. [28]	51/51	-
Krahl & Sellheyer [29]	28/28	-
Ishida, et al. [30]	20/20	-
Filho, et al. [31]	20/20	-
Mashhood, et al. [32]	29/29	0/11
Tan & Sunjaya [9]	23/23	-
Kirzhner & Jakobiec [33]	13/13	-
Dasgeb, et al. [24]	24/24	1/11 (weak positive)
Patil, et al. [12]	9/9	-
Sellheyer, et al. [34]	17/17	0/75
Ansai, et al. [35]	30/31	-
BerEP4 positive	284	1
Ber EP4 negative	1	116

Total	**285**	**117**

Sensitivity	284/285 × 100 = **99.6%**	
Specificity	116/117 × 100 = **99.2%**	
Positive predictive value	284/285 × 100 = **99.6%**	
Negative predictive value	116/117 × 100 = **99.1%**	

**Table 2:** Sensitivity and specificity of BerEP4 in differentiating BCC with the epithelial disorders below as control.

Epithelial skin disorder	Sensitivity (%)	Specificity (%)
Actinic keratosis	100 [36]	100 [36]
	100 [24]	100 [24]
Seborrheic keratosis	97 [35]	100 [35]
	100 [24]	100 [24]
Poroma [35]	97	100
Lichen planus like keratosis [24]	100	100
Nevi [24]	100	100
Hemangioma [24]	100	100
Inverted follicular keratosis [24]	100	100
Squamous intraepithelial neoplasia (SIN) [36]	100	100
Sebaceous adenoma/hyperplasia [24]	100	83.3
